# Hand Washing Induces a Clean Slate Effect in Moral Judgments: A Pupillometry and Eye-Tracking Study

**DOI:** 10.1038/srep10471

**Published:** 2015-05-21

**Authors:** Kai Kaspar, Vanessa Krapp, Peter König

**Affiliations:** 1 Social and Media Psychology, Department of Psychology, University of Cologne, Cologne, Germany; 2 Neurobiopsychology, Institute of Cognitive Science, University of Osnabrück, Osnabrück, Germany; 3 Department of Neurophysiology and Pathophysiology, University Medical Center Hamburg-Eppendorf, Hamburg, Germany

## Abstract

Physical cleansing is commonly understood to protect us against physical contamination. However, recent studies showed additional effects on moral judgments. Under the heading of the “Macbeth effect” direct links between bodily cleansing and one’s own moral purity have been demonstrated. Here we investigate (1) how moral judgments develop over time and how they are altered by hand washing, (2) whether changes in moral judgments can be explained by altered information sampling from the environment, and (3) whether hand washing affects emotional arousal. Using a pre-post control group design, we found that morality ratings of morally good and bad scenes acquired more extreme values in the control group over time, an effect that was fully counteracted by intermediate hand washing. This result supports the notion of a clean slate effect by hand washing. Thereby, eye-tracking data did not uncover differences in eye movement behavior that may explain differences in moral judgments. Thus, the clean slate effect is not due to altered information sampling from the environment. Finally, compared to the control group, pupil diameter decreased after hand washing, thus demonstrating a direct physiological effect. The results shed light on the physiological mechanisms behind this type of embodiment phenomenon.

The fundamental goal of physical cleansing is to protect us against physical contamination and, hence, to foster physiological health. However, empirical studies showed that physical cleansing also touches psychological aspects. Already, Shakespeare described in his drama that Lady Macbeth repeatedly experienced the desire to wash her hands after she incited her husband to kill the King of Scotland. Accordingly, Zhong and Liljenquist^1^ found that moral impurity actually elicits the desire for cleansing-related products. This so-called “Macbeth effect” stimulated further research on the relationship between physical and moral purity. In a follow-up study, they demonstrated that washing one’s hands after recalling an unethical deed of the past reduced the motivation to volunteer, thus indicating that hand washing already restored a suitable moral self-image and, hence, reduced the desire to compensate the unethical deed by voluntary help. More recently, Reuven *et al.*^2^ replicated this finding, while the effect was stronger in participants with obsessive-compulsive disorder. In a study by Gollwitzer and Melzer^3^, inexperienced video game players felt higher moral distress after playing a video game that included violence against humans (versus objects), and they selected more hygiene products in a subsequent product choice task than frequent video game players. Moreover, in a study by Denke *et al.*^4^, participants enacted a sequence of scenarios that included either a moral deed (telling the truth) or an immoral one (lying). Immediately after a scenario ended, they rated the desirability of different products, while only cleansing-related products were rated as more desirable after an immoral act than after a moral one. At the same time, this Macbeth effect was accompanied by an active cortical network that comprises sensorimotor brain areas, thus indicating an embodiment of the moral-purity metaphor. Also, Lobel *et al.*^5^ found that more money was donated to charity before rather than after bathing for religious purification. Finally, Lee and Schwarz^6^ found that the Macbeth effect seems to be specific with respect to the motor modality that is primarily involved in the moral transgression. The desire for mouthwash was high after communicating a lie via voice mail, while the desire for hand sanitizer was higher after lying in an email. All these observations demonstrate a direct link between one’s own bodily cleanliness and moral purity. Physical cleansing seems to have the power to restore the perception of one’s own moral self that, in turn, may reduce or even eliminate the Macbeth-like cognitions and behavior.

Research on embodied cognition provides a simple explanation for this effect: Williams *et al.*^7^ stated that “early sensorimotor experiences serve as the foundation for the later development of more abstract concepts and goals” (p. 1257). In early childhood, we learn the concept of physical purity and how to achieve this goal. This knowledge serves as the scaffold for the later development of conceptually related abstract ideas such as moral purity. Accordingly, Lakoff and Johnson^8^ proposed in their work on conceptual metaphors that abstract concepts are built upon earlier experiences that are often body-related. As a consequence, established associations between the physical and abstract cognitive concept emerge that are assumed to be stable across the whole lifespan^9^ and are presumably grounded on the same neuroanatomical foundation^10^. Similarly, according to Rozin’s body-to-soul-preadaptation theory of disgust^11^, a neuronal circuitry, developed for the processing of the originally food-related emotion of disgust, was preadapted for an extension to other threats beyond the rejection of food, such as moral threats. Indeed, Schnall *et al.*^12^ found that the feelings of disgust increased the severity of moral judgments relative to controls. Correspondingly, Moll *et al.*^13^ found that strongly overlapping brain regions were activated by pure disgust (without moral connotations) and the moral emotion of indignation. The strong conceptual and neuroanatomical relationship between moral and physical purity might explain why physical cleansing can affect moral reasoning and vice versa.

Importantly, the psychological effect of physical cleansing is not limited to the moral domain. Recent studies showed that washing one’s hands can wash off traces of the past in general. Lee and Schwarz^14^ found that washing can reduce post-decisional dissonance effects and Xu *et al.*^15^ showed that the experience of bad luck and good luck can be washed away. Kaspar^16^ found that hand washing after failure in an anagram task did not only increase optimism but also hampered future performance. The author assumes that hand washing may have “lowered participants’ need to work harder in order to restore their perception of competence” (p. 71). Finally, Florack *et al.*^17^ found that hand washing can reduce decision preferences that are biased by ownership. Given these findings, the Macbeth effect may only be one signature of a more general “clean slate effect,” as termed by Lee and Schwarz^14^. Correspondingly, Xu *et al.*^15^ concluded that hand washing removes both desirable as well as undesirable traces of the past. Similarly, Florack *et al.*^17^ stated that “the physical action of hand washing resets the cognitive system to a more neutral state” (p. 288). Hence, hand washing apparently does not only help to wash away our sins^1^ but also has the power to generally attenuate the effects of past events.

However, in addition to the idea of a general clean slate effect, it has been shown that hand washing, as well as the mere activation of the cleanliness concept, can alter moral judgments. This aspect is crucial, as it includes the question of how cleanliness treatments work in a specific context. Zhong *et al.*^18^ reported that study participants who had either actually cleansed their hands or merely visualized themselves in a clean versus dirty state rated critical social issues, such as pornography and smoking, to be more morally wrong. Also, participants who were primed for cleanliness reported a higher moral self-image that mediated the effect of the cleanliness priming on moral judgments. This result indicates that the attribution of physical purity to oneself generalizes to the impression of moral purity as well. Consequently, when we feel to be morally clean, this may license harsher moral judgments.

In contrast, Schnall *et al.*^19^ found that certain moral actions were judged less severe, in contrast to a control condition, when the concept of physical cleanliness was activated either by a sentence unscrambling task or by washing one’s hands after experiencing disgust. Zhong *et al.*^18^ proposed a simple explanation for the seemingly contradicting results; in their study, the activation of the cleanliness concept apparently restored one’s own moral self-image, and in the work of Schnall *et al.*^19^, the state of cleanliness was perhaps attributed to the judged target but not to the self of the judging person. Hence, it seems to be crucial who is viewed as clean. Johnson *et al.*^20^ failed to replicate the results of Schnall *et al.*^19^, but in a more recent replication study, Huang^21^ showed that less severe moral judgments only occurred when study participants invested low effort in the sentence unscrambling task. We suppose that low task involvement facilitated the attribution of cleanliness to the judged targets.

To conclude, activating cleanliness cognitions with a priming procedure showed mixed effects that are presumably signatures of different attribution processes. In contrast, washing one’s own hands makes the attribution of a clean state to oneself indubitable. Thereby, two mechanisms have been posited so far: on the one hand, washing may lead to an increase in one’s perceived moral purity and, hence, makes judgments about others’ moral transgressions more severe. On the other hand, washing may lead to a general clean slate effect, thus attenuating the impression of past events and setting the cognitive system to a more neutral state. The present study contrasts these two potential mechanisms in the moral domain by changing the experimental paradigm and methodology.

## The Present Study

In contrast to previous studies on cleanliness effects, we applied a pre-post control group design. Hitherto, it is unclear how moral judgments evolve across several issues of similar morality. Smith *et al.*^22^ found that increasing the familiarity of information about a person leads to less systematic or analytic processing of this information, thus resulting in more stereotyped judgments and less individualized judgments. Correspondingly, we assume that stereotyping in the moral domain would be reflected by more extreme judgments when judging numerous morally good and bad actions in a sequence. In such a situation, the notion of a clean slate effect suggests that hand washing in the meantime would counter increasing stereotyping, thus leading to less extreme judgments after hand washing due to a reset of the cognitive moral system. In contrast, the notion of an enhanced moral self by cleansing one’s hands would predict even more severe moral judgments. However, previous studies have mainly focused on immoral actions and, hence, the question arises as to whether the effect of hand washing would also generalize to morally good issues. Indeed, according to the idea of moral consistency, moral self-regard may motivate to behave in a way that helps to maintain a high moral self^5^. In this sense, hand washing may also lead to more extreme judgments in the case of morally good actions in order to perpetuate one’s high moral self by emphasizing the praiseworthiness of moral actions.

Moreover, we asked whether hand washing modulates information sampling or whether it affects moral judgements at a later stage of the judgment process. Two extreme scenarios are conceivable. The act of hand washing could alter sampling information from the environment. The unchanged process of moral judgement would act on a different set of sampled information and potentially reach a different verdict. Alternatively, the act of hand washing could be neutral with respect to the sampling of information, but the process of moral judgement would evaluate identical information differently. Only the latter case would exemplify a direct link between hand washing and moral judgments. In fact, we propose to interpret the effects of washing in terms of information sampling and weighting. The hitherto reported effects showed that the act of cleansing did not literally wash away what had already happened in the past. Instead, washing led to a specific (re-)weighting of the available information. For example, hand washing did not undo an unethical deed that participants had previously committed^1^, did not change previous decisions^14^, and did not substitute a failure experience by success^16^, but it changed the weighting of unethical deeds, reduced the need to devaluate non-chosen options, and increased one’s optimism to be more successful in the future, respectively. In each of these cases, we can assume that the effect was based on a selective sampling of information. In fact, as highlighted by Lindskog *et al.*^23^, “several accounts of human judgment and decision making assume that people sample information from memory prior to making a judgment or decision” (p. 783). However, we do not only selectively sample information from memory but also from the environment, while the current information sampling depends on the individual’s attention, capacity, and processing goals^24^. Thus, the effects of hand washing may base on sampling biases on both stages. Following Smith *et al.*^22^, we expected that increasing the familiarity of information (induced by a long sequence of moral vs. immoral scenes) would lead to a less systematic processing of information and result in more stereotyped (i.e., extreme) moral judgments (see above). If hand washing affects information sampling from memory (e.g., when classifying a specific scene as very immoral compared to social norms stored in memory), it should not show an effect on the visual exploration of moral and immoral scenes. If, however, hand washing affects information sampling from the environment, changes on the level of eye movements should be traceable. According to the notion of a clean slate effect, washing should reset one’s cognitive state to a more neutral one^17^ and, hence, increase peoples’ openness for each individual scene so that they are more motivated to visually explore it before making a moral judgment. Correspondingly, Ybarra^25^ stated that “being cognitively open refers to the tendency to seek out and integrate additional information into a current judgment” (p. 431). In fact, when people repeatedly observe visual scenes, their viewing activity decreases, while higher interest in visual scenes elicits a more active exploration of the scenes^26^. In contrast, if washing elicits the impression of one’s own moral cleanliness, even more severe moral judgments are conceivable, which could result from an additionally lowered engagement in stimulus exploration.

Finally, does hand washing affect the physiological level or is its impact limited to purely disembodied cognitive processes? This is an important question, as the majority of results rests on self-reports by the experimental subjects being susceptible to many external factors. Previous studies exclusively focused on the self-reported emotional effects of cleanliness and provided mixed results. While Liljenquist *et al.*^27^ found no effect of a clean-scented room on positive and negative affect, Zhong and Liljenquist^1^, as well as Reuven *et al.*^2^, found reduced negative emotions by hand washing after recalling an unethical deed of the past. According to the clean slate effect of washing, the level of participants’ arousal should also be reset to a more neutral state.

To summarize, the present study addresses the three questions described above: (1) we used a pre-post control group design to investigate whether hand washing would cushion increasing stereotyping in moral judgments, as suggested by Smith *et al.*^22^. Accordingly, we hypothesized that the difference in morality ratings for moral versus immoral scenes increases over time. Thereby, hand washing may induce a generalized “clean slate effect”^14^,^15^ for immoral as well as moral judgments that counteract this tendency or, alternatively, washing may elicit even more extreme judgments due to the impression of an enhanced moral self-image^18^ (Hypothesis 1, Change in Morality Ratings). (2) We applied eye-tracking methodology to clarify whether the sampling and processing of information is actually influenced by hand washing, thus providing an objective measurement. This allows testing of the hypothesis that the impact of hand washing is limited to the formation of moral judgments and does not touch early processes of information gathering (Hypothesis 2, Information Sampling). (3) We tracked pupil size before and after hand washing as an objective measurement for subjects’ emotional arousal^28^. If the clean slate effect of hand washing also affects physiological arousal, pupil diameter should decrease after hand washing (Hypothesis 3, Physiological Effects).

## Methods

All the participants gave written informed consent to participate in this study. We performed the study in accordance with the Declaration of Helsinki and national guidelines of the German Psychological Society. The experimental methods were approved by the Ethical Committee of the University of Osnabrück (Germany).

### Participants

Forty subjects (16 males), who were naive to the purpose of the study, participated for course credits. Their average age was 22.25 years (*SD* = 3.85). We selected the number of subjects according to previous studies in the field that reported significant differences between a real cleansing condition and a control condition in which no hand cleansing was applied. Across four studies,^14^ (Study 1;^15^ Study 1 and 2;^19^ Study 2), a sample size of *n* = 20 per condition was found to be sufficient. All subjects had normal or corrected-to-normal visual acuity and passed the Ishihara Test for Color Blindness^29^. They were randomly assigned to one of the two experimental conditions (*washing group* vs. *no washing group*).

### Material and Apparatuses

The subjects observed 72 complex images, 36 immoral scenes, and 36 moral scenes. The immoral scenes were selected in accordance to the morality ratings of different negative social issues^18^, and the moral scenes were selected according to the notion that morally good actions are those that bring about a great amount of happiness and the least amount of physical or psychological pain for other persons or the environment. The scenes depicted human behavior that is either morally bad (e.g., physically beating someone up, polluting the environment, stealing something, taking illegal drugs) or good (e.g., helping old people, handing a gift, caring for children, laughing together). The images were partially borrowed from the International Affective Picture Set (IAPS,^30^) and partially borrowed from the internet. All images had a resolution of 1280 × 960 and were full-colored. Two independent raters categorized all images and completely agreed. Inter-rater agreement was additionally validated by a sample of 20 subjects (not included in the eye-tracking session) who observed all images in a randomized order and rated each image on a 9-point scale from 1 (*very immoral*) to 9 (*very moral*). On average, all 36 immoral scenes were rated ≤3.95 (*SD* = 1.79), and all 36 moral scenes were rated ≥5.60 (*SD* = 1.57).

Images were presented on a 21-in. Samsung SyncMaster 1100 DF 2004 CRT Monitor (Samsung Electronics, Seoul, South Korea). The display resolution was 1280 × 960 pixels, the refresh rate was 85 Hz, and the distance to the screen was set at 80 cm without a headrest to facilitate normal viewing conditions. The computer running the experiment was connected to the host computer (Pentium 4, Dell Inc., Round Rock, TX, USA) with EyeLink software via a local network.

An EyeLink II system (SR Research, Ontario, Canada) recorded subjects’ pupil size and eye movements at a sampling rate of 500 Hz and compensated for head movements. In order to calibrate the system, the subjects made saccades to a grid of 13 fixation spots on the screen, which appeared one by one in a random order. The tracking of the eye, which provided the lower validation error, started as soon as this value was below the 0.5° visual angle. A fixation spot appeared after each trial in the middle of the screen in order to control for slow drifts in measured eye movements. In the cases of an error larger than 1°, calibration and validation were repeated.

### Procedure

Upon arrival, the subjects were introduced into the study procedure and had to sign a consent form. As a recent study revealed the strong impact of mood on how people observe complex images^31^, the subjects initially rated their current mood on four 5-point Likert-items to assess their current happiness, alertness, calmness, and listlessness^32^. This measurement enabled us to check whether potential differences in the dependent variables between the washing and non-washing group were contaminated by pre-experimental differences in subjects’ mood. No difference was revealed, all *t*(38) ≤ 1.29, *p* ≥ 0.206. Afterward, the subjects sat down in front of a screen. The experimenter instructed them to freely observe each of the following images and to subsequently rate the morality of the depicted scene by a key press. Then, the eye-tracker was calibrated and validated. Each of the 72 scenes was presented for six seconds in accordance to previous studies (e.g.,^26^,^31^,^33^) and were followed by a screen that asked to rate the morality of the previous scene on a 9-point scale from 1 (*very immoral*) to 9 (*very moral*). The next scene was presented after a corresponding key press. After the subjects had observed half of the scenes (block 1), a break was introduced. The subjects were told that they had to perform a sensorimotor coordination task in order to maintain concentration and attention for the second part of the eye-tracking session. The subjects played Jenga for two minutes, but half of them previously washed their hands, allegedly due to hygienic reasons (*washing group*), whereas the control group (*no washing group*) waited for an additional two minutes. After the break, the subjects continued with scene observation and evaluation (block 2). The sequence of scenes was constant across the subjects in favor of the between-subject comparison (washing vs. no washing group). Randomized scene sequences usually produce additional noise in the eye-tracking data, and they do not counterbalance the potential priming effects in the viewing behavior from one scene to the next (cf.^31^). However, the order of scenes was pseudo-randomized within the subjects; that is, three of six successively presented scenes depicted immoral content, while the next immoral scene was not predictable.

Moreover, as we were interested in a within-subject effect of hand washing between the first and second half of the eye-tracking session, we ensured that both subsets of scenes (18 immoral and 18 moral scenes before as well as after the break) did not differ in visual saliency. Therefore, each of the 72 scenes was analyzed by means of a saliency map computation. The standard algorithm^34^ predicts the fixation patterns by a decomposition of pictures into several physical features (e.g., contrast and orientation). Additionally, we applied a more recent graph-based visual saliency algorithm (GBVS) to predict the fixations with an even higher reliability^35^. Both algorithms revealed no difference between the scenes of the first and second eye-tracking part, both *t*(35) ≤ 0.10, *p* ≥ 0.924.

Finally, in order to check whether the image ratings depended on the specific image sequence in the eye-tracking session, a third group (*comparison group*) of 20 subjects with a mean age of 20.75 (*SD* = 2.65) observed and rated all images in a randomized order (without eye-tracking and an intermediate break).

### Eye Movement Parameters

In order to appropriately describe the viewing behavior of study participants, we selected parameters that are both very sensitive to the emotional and motivational states of the observer as well as a suitable indicator for global viewing activity^26^,^31^. In accordance with previous studies on human vision, we therefore focused on the mean length of saccades as an indicator for the visual step size that participants use to scan the scenes. The detection of a saccade was based on the following three default measures: amplitude of at least 0.1° visual angle, with a velocity of at least 30°/s, and with an acceleration of at least 8000°/s^2^. The minimal saccade velocity was 25°/s after saccade onset. All these values had to be sustained for at least 4 ms for a valid saccade. In contrast, fixations were defined as periods without saccades^36^. We also investigated the mean duration of fixations because it is a sensitive indicator for information processing depth. It is believed that longer fixation durations indicate a deeper and more extensive processing of visual stimuli^37^,^38^. The eye-tracker detected and automatically parameterized fixations and saccades during each fixation. The first fixation of each trial was excluded from the analysis because its localization was determined by the preceding fixation spot used for drift correction. Finally, we applied an entropy measurement to quantify the extent of the spatial exploration of the scenes. Entropy has the advantage of measuring the spread of fixation distributions without the necessity to make assumptions about scene-specific geometrical arrangements. Higher entropy values indicate a more extensive fixation distribution; that is, a more spatially extended exploration of a scene. Thereby, the absolute values in entropy are negligible, as they depend on image resolution and the size of the Gaussian kernel that we used to convolve the fixation distribution map (leading to a fixation density map that is used for entropy calculation). This procedure is described in detail elsewhere^39^.

## Results

### Hypothesis 1: Change in Morality Ratings

**Figure 1:**
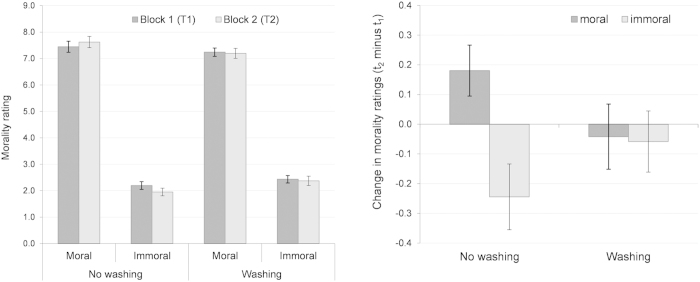
The effect of hand washing on morality ratings. The left panel depicts absolute values, the right panel depicts the change in morality ratings from block 1 to block 2 by means of difference score*s* (t_2_ minus t_1_). Vertical lines indicate the standard error of the mean.

We initially calculated an IMAGE SET (moral vs. immoral scenes) x TIME POINT (before vs. after the break) x WASHING CONDITION (washing vs. no washing during the break) mixed-measures ANOVA (Greenhouse-Geisser applied). As expected, we found a significant effect of the image set on the morality ratings, *F*(1, 38) = 703.67, *p* < 0.001, *η*_*p*_^*2*^ = 0.95, with much higher ratings for the moral images (*M* = 7.38, *SD* = 0.84) than for the immoral images (*M* = 2.24, *SD* = 0.66) (see Fig. 1). Moreover, we found an interaction between the image set and time point, *F*(1, 38) = 4.93, *p* = 0.032, *η*_*p*_^*2*^ = 0.12, a marginally significant interaction between image set and washing condition, *F*(1, 38) = 2.78, *p* = 0.104, *η*_*p*_^*2*^ = 0.07, as well as a three-way interaction, *F*(1, 38) = 4.21, *p* = 0.047, *η*_*p*_^*2*^ = 0.10. No further effects occurred, all *F*(1, 38) ≤ 0.60, *p* ≥ 0.444, *η*_*p*_^*2*^ ≤ 0.02. Because we were primarily interested in the potential within-subject changes between the first and second block of the experiment, we subsequently investigated whether the morality ratings changed within the *washing group* and the *no washing group*. In fact, the *no washing group* showed more extreme morality ratings in block 2 (t_2_) than in block 1 (t_1_) in both directions. The ratings for the moral scenes increased from the first to the second block, *t*(19) = −2.11, *p* = 0.049, *d* = 0.47, while the ratings for the immoral scenes decreased, *t*(19) = 2.21, *p* = 0.040, *d* = 0.49. In contrast, in the *washing group,* neither the morality ratings of the moral scenes changed nor the morality ratings for the immoral images, both *t*(19) ≤ 0.57, *p* ≥ 0.578, *d* ≤ 0.13.

To make this interaction more visible, we computed the difference scores between t_2_ (block 2) and t_1_ (block 1), which served as a dependent variable in a 2 × 2 (IMAGE SET x WASHING CONDITION) ANOVA. As shown in Fig. 1, we again found no main effect of the washing condition, *F*(1, 38) = 0.03, *p* = 0.866, *η*_*p*_^*2*^ < 0.01, but the difference scores revealed a significant main effect of the image set, *F*(1, 38) = 4.93, *p* = 0.032, *η*_*p*_^*2*^ = 0.12, with a negative difference score for the moral scenes, but a positive difference score for the immoral scenes. This main effect reflects the image set by the time point interaction found in the initial three-way ANOVA. Moreover, the image set interacted with the washing condition, *F*(1, 38) = 4.21, *p* = 0.047, *η*_*p*_^*2*^ = 0.10, thus reflecting the three-way interaction previously found.

Finally, in order to verify that the tendency toward more extreme morality ratings in the *no washing group* was not an artefact of the constant sequence of scenes, we tested the *comparison group,* in which the image sequence was randomized across subjects. In this group, the difference scores for the moral and immoral scenes (mean of last 36 images vs. first 36 images) also differed significantly, *t*(19) = 2.38, *p* = 0.028, *d* = 0.53, with increased morality ratings for moral scenes (+0.158), but slightly decreased ratings for immoral scenes (−0.053).

To conclude, on a scale ranging from *very immoral* to *very moral,* we found much higher morality ratings for the moral scenes than for the immoral scenes, thus validating our stimulus set. Even more importantly, we found more stereotyped morality judgments over time (block 2 vs. block 1), but this effect disappeared when participants washed their hands during the break. Thus, the results support the notion of a clean slate effect. Hand washing apparently reset the dynamic in participants’ morality judgments.

### Hypothesis 2: Information Sampling

**Figure 2:**
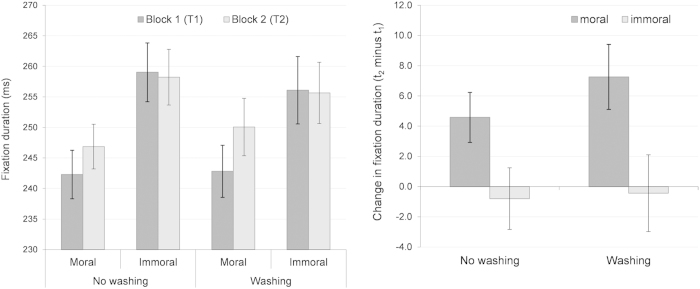
The mean duration of fixations. The left panel depicts absolute values, the right panel depicts the change in fixation duration from block 1 to block 2 by means of difference score*s* (t_2_ minus t_1_). Vertical lines indicate the standard error of the mean.

With respect to the mean duration of fixations, we initially calculated a 2 × 2 × 2 (IMAGE SET x TIME POINT x WASHING CONDITION) ANOVA. As shown in Fig. 2, we found a significant main effect of the image set, *F*(1, 38) = 103.60, *p* < 0.001, *η*_*p*_^*2*^ = 0.73, with longer fixations on the immoral versus moral scenes. We also found a main effect of the time point, *F*(1, 38) = 4.56, *p* = 0.039, *η*_*p*_^*2*^ = 0.11, with longer fixations in block 2 versus block 1. The image set and time point also significantly interacted, *F*(1, 38) = 15.08, *p* < 0.001, *η*_*p*_^*2*^ = 0.28, whereby the effect of the time point (i.e., longer fixation durations in block 2 vs. block 1) was only present on moral scenes, *t*(39) = 4.36, *p* < 0.001, *d* = 0.69, but not on immoral scenes, *t*(39) = −0.38, *p* = 0.70, *d* = 0.06. The ANOVA did not show further significant effects, all *F*(1, 38) ≤ 0.47, *p* ≥ 0.495, *η*_*p*_^*2*^ ≤ 0.01, except a just significant interaction between image set and washing condition, *F*(1, 38) = 4.00, *p* = 0.053, *η*_*p*_^*2*^ = 0.10, with a higher difference between immoral and moral scenes in the *no washing group* versus *washing group*. However, and importantly, this slight interaction has to be interpreted with caution. Although the three-way interaction did not reach statistical significance, it is not apparent how much of the group difference is due to between-subject differences that already existed from the outset; that is, before the cleanliness treatment was applied to the washing group. Thus, to clarify whether hand washing actually affected participants’ fixation durations, we again calculated the difference scores between the two blocks (t_2_ minus t_1_) that represent the temporal change and thus correct for the group differences that might have already existed before the cleanliness treatment. We used the difference scores as a dependent variable in a 2 × 2 (IMAGE SET × WASHING CONDITION) ANOVA. We found no effect of the washing condition and no interaction, both *F*(1, 38) ≤ 0.47, *p* ≥ 0.495, *η*_*p*_^*2*^ ≤ 0.01, but the difference in the fixation duration between block 1 and block 2 differed between the moral and immoral scenes, *F*(1, 38) = 15.08, *p* < 0.001, *η*_*p*_^*2*^ = 0.28, thus reflecting the image set by time point interaction reported above. As shown by Fig. 2, in both experimental groups, the fixation durations on moral scenes were longer after the break, both *t*(19) = 2.76, *p* ≤ 0.012, *d* ≥ 0.62, while there was no difference between the two groups, *t*(38) = 0.99, *p* = 0.330, *d* = 0.31. Regarding the immoral scenes, the difference scores neither differed from zero within the two groups, both |*t*|(19) ≤ 0.39, *p* ≥ 0.700, *d* ≤ 0.09, nor between them, *t*(19) = 0.11, *p* = 0.912, *d* = 0.04. Consequently, hand washing did not significantly affect the duration of fixations.

**Figure 3:**
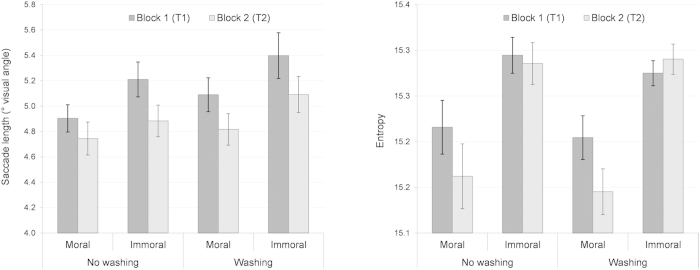
The mean length of saccades (left panel) and the mean entropy quantifying the extent of spatial exploration of scenes (right panel). Vertical lines indicate the standard error of the mean.

In the next step, we investigated the length of saccades that participants made to scan the scenes. The 2 × 2 × 2 (IMAGE SET x TIME POINT x WASHING CONDITION) ANOVA showed a main effect of the time point, *F*(1, 38) = 23.71, *p* < 0.001, *η*_*p*_^*2*^ = 0.38, with shorter saccades in block 2 versus 1 (see Fig. 3). Moreover, a main effect of the image set occurred, *F*(1, 38) = 31.42, *p* < 0.001, *η*_*p*_^*2*^ = 0.45, with longer saccades on immoral versus moral scenes. No further effects were found, *F*(1, 38) ≤ 2.05, *p* ≥ 0.160, *η*_*p*_^*2*^ ≤ 0.05. Accordingly, when using difference scores (t_2_ minus t_1_) in a 2 × 2 (IMAGE SET x WASHING CONDITION) ANOVA, no effect remained, all *F*(1, 38) ≤ 2.05, all *p* ≥ 0.160, all *η*_*p*_^*2*^ ≤ 0.05, because calculating the difference scores naturally eliminated the main effects of the time point and image set.

Finally, we investigated the extent of the spatial exploration of images quantified by entropy. The 2 × 2 × 2 (IMAGE SET × TIME POINT × WASHING CONDITION) ANOVA again showed a main effect of the time point, *F*(1, 38) = 9.13, *p* = 0.004, *η*_*p*_^*2*^ = 0.19, a main effect of the image set, *F*(1, 38) = 79.62, *p* < 0.001, *η*_*p*_^*2*^ = 0.68, and an interaction between these two factors, *F*(1, 38) = 20.65, *p* < 0.001, *η*_*p*_^*2*^ = 0.35. As depicted in Fig. 3, the subjects explored moral scenes more spatially extensively in block 1 versus block 2, *t*(39) = 5.06, *p* < 0.001, *d* = 0.80, while the immoral scenes were equally extensively explored before versus after the break, *t*(39) = −0.302, *p* = 0.765, *d* = 0.05. The ANOVA did not show further significant effects, all *F*(1, 38) ≤ 1.29, all *p* ≥ 0.264, all *η*_*p*_^*2*^ ≤ 0.03.

To sum up, the viewing behavior changed between block 1 and block 2 in terms of longer fixations on the moral (but not immoral) scenes, which were paralleled by a less spatially extensive exploration (entropy). Moreover, saccades were longer before versus after the break, and they were longer on immoral versus moral scenes. However, and in contrast to the morality judgments, hand washing did not influence eye movement parameters. Consequently, the impact of hand washing did not touch early processes of information sampling from the environment.

### Hypothesis 3: Physiological Effects

**Figure 4:**
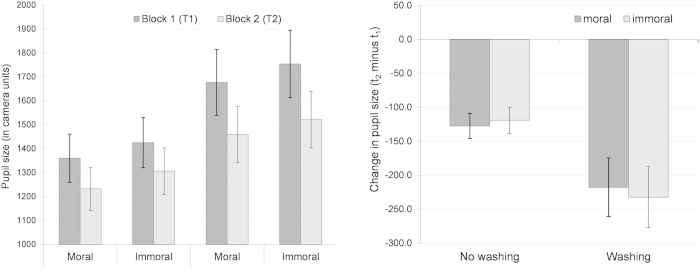
The effect of hand washing on pupil size. The left panel depicts absolute values, the right panel depicts the change in pupil size from block 1 to block 2 by means of difference scores (t_2_ minus t_1_). Vertical lines indicate the standard error of the mean.

We initially calculated a 2 × 2 × 2 (IMAGE SET × TIME POINT × WASHING CONDITION) ANOVA with pupil size as the dependent variable. As shown by Fig. 4, we found a significant main effect of the image set, *F*(1, 38) = 196.51, *p* < 0.001, *η*_*p*_^*2*^ = 0.84, with larger pupil sizes when observing immoral versus moral scenes. We also found a main effect of the time point, *F*(1, 38) = 53.91, *p* < 0.001, *η*_*p*_^*2*^ = 0.59, with larger pupil sizes in block 1 versus block 2. Moreover, we found a marginally significant main effect of the washing condition, *F*(1, 38) = 2.89, *p* = 0.097, *η*_*p*_^*2*^ = 0.07, with larger pupil sizes in the washing versus no washing group. Also, we found an interaction between the time point and washing condition, *F*(1, 38) = 4.61, *p* = 0.038, *η*_*p*_^*2*^ = 0.11. The three-way interaction was marginally significant, *F*(1, 38) = 2.78, *p* = 0.104, *η*_*p*_^*2*^ = 0.07. No interaction between image set and washing condition, as well as between image set and time point, occurred, both *F*(1, 38) ≤ 0.22, *p* ≥ 0.643, *η*_*p*_^*2*^ ≤ 0.01.

In order to further scrutinize the interactions with regard to the impact of the washing treatment, we again computed the difference scores (t_2_ minus t_1_) serving as the dependent variable in a 2 × 2 (IMAGE SET × WASHING CONDITION) ANOVA. This analysis revealed no main effect of the image set, *F*(1, 38) = 0.22, *p* = 0.643, *η*_*p*_^*2*^ = 0.01, but a marginally significant interaction, *F*(1, 38) = 2.78, *p* = 0.104, *η*_*p*_^*2*^ = 0.07, that reflected the three-way interaction reported above. Finally, the washing condition showed a significant main effect, *F*(1, 38) = 4.61, *p* = 0.038, *η*_*p*_^*2*^ = 0.11, that reflected the washing condition by time point interaction reported above. As Fig. 4 shows, in both conditions (washing and no washing), the difference in pupil size was significantly negative (one-sample t-tests against zero: all *p* < 0.001), thus indicating a smaller pupil size after the break. However, the decrease in pupil size was significantly larger when subjects had washed their hands between block 1 and block 2. Consequently, hand washing also affected the physiological level.

## Discussion

Hand washing is a daily ritual whose fundamental goal is to protect us against physical and biological contamination and, hence, foster physiological health. However, empirical evidence showed that hand washing also affects moral judgments. Unmoral deeds enhance the desire for cleansing products in terms of a Macbeth effect^1^,^3^,^4^, and the impact of washing goes beyond judgment formation, as it induces a kind of clean slate effect in several domains^14^,^15^,^16^,^17^. The present study addresses three critical questions focused on the original domain of moral judgments.

First, we found that hand washing between the two blocks induced a clean slate effect in the form of eliminating the tendency for more extreme moral judgments over time. This finding supports previous studies that reported clean slate effects regarding cognitive dissonance^14^, good and bad luck^15^, failure experience^16^, and product evaluation^17^. In each case, physical cleansing washed away traces of the past by resetting previous experiences. The present within-subject design revealed such a general clean slate effect in the domain of moral judgments, thus generalizing to the judgment of other people’s bad and good deeds (Hypothesis 1).

Second, we found more extreme moral judgments over time for moral and immoral scenes as long as no hand washing took place in the meantime. This finding corresponds to the results of Smith *et al.*^22^, who found that increased familiarity of information fosters stereotyped judgments. Smith *et al.* explained this by changes in information processing. According to their findings, increasing familiarity of information leads to less systematic or analytic processing, thus resulting in more stereotyped judgments. Indeed, we found changes in viewing activity as a signature of image processing from the first to the second block. This effect was, however, mainly limited to the moral scenes: fixation duration increased and the amount of spatial exploration decreased on the moral images over time while no change occurred on the immoral scenes. Moreover, saccades were shorter in block 2 versus 1, and they were longer on immoral versus moral scenes. This result pattern matches studies showing that images of negative valence are observed more actively^31^. Beyond this, we found no hint that hand washing affects eye-tracking parameters. This result is evidence against an effect of physical cleansing on information sampling from the environment. Instead, the effect of hand washing seems to affect the selection and/or weighting of information stored in memory (e.g., social norms).

Third, the present study provides objective evidence for an impact of physical cleansing on the physiological level. While previous studies limited the focus to self-reports, we analyzed pupil size as an indicator for emotional arousal^28^. We found that pupil size decreased from the first to the second measurement block, while hand washing in between the measurement blocks additionally decreased pupil size (Hypothesis 3). Presumably, the subjects successively habituated to the (im)moral scenes indicated by a downregulation of arousal. The immoral scenes are especially arousing, as shown by the norm values of the IAPS manual^30^. Hand washing seems to facilitate emotional downregulation, as also suggested by Zhong and Liljenquist^1^, as well as Reuven *et al.*^2^, who found attenuated reports of negative emotions when subjects washed their hands after they had recalled an unethical deed of the past. Consequently, the clean slate effect is more than a cognitive reset, and hand washing actually affects the physiological level.

Given the present findings, future studies in the field of embodied cognition research should fathom the psychophysiological effects of phenomena that have been primarily investigated on the behavioral level so far – such as physical cleansing. A bulk of studies has already shown that the effects of bodily sensations are not limited to cognitive processes but they also affect emotional states (cf.^10^,^40^). The effect of hand washing on pupil size and the unaffected eye movement behavior indicate a fruitful avenue for follow-up studies. Uncovering the psychophysiological mechanisms behind embodiment phenomena not only increases our understanding of the cognitive consequences of bodily sensations such as morality judgments. It also helps to exploit the potential for therapeutic and other applied purposes.

## Additional Information

**How to cite this article**: Kaspar, K. *et al.* Hand Washing Induces a Clean Slate Effect in Moral Judgments: A Pupillometry and Eye-Tracking Study. *Sci. Rep.* **5**, 10471; doi: 10.1038/srep10471 (2015).
